# Identification of candidate SNPs associated with embryo mortality and fertility traits in lactating Holstein cows

**DOI:** 10.3389/fgene.2024.1409335

**Published:** 2024-08-09

**Authors:** Carolina L. Gonzalez Berrios, Courtney F. Bowden, Hamad M. Saad, Jeanette V. Bishop, Hana Van Campen, Pablo Pinedo, Thomas R. Hansen, Milton G. Thomas

**Affiliations:** ^1^ Animal Reproduction and Biotechnology Laboratory, Department of Biomedical Sciences, Colorado State University, Fort Collins, CO, United States; ^2^ Department of Animal Sciences, College of Agricultural Sciences, Colorado State University, Fort Collins, CO, United States; ^3^ Texas A&M AgriLife Research Station, Beeville, TX, United States

**Keywords:** cattle, health traits, pregnancy, Holstein cows, early embryo mortality, single-nucleotide polymorphisms, linkage disequilibrium

## Abstract

**Introduction:** Targeted single nucleotide polymorphisms (SNPs) have been used in genomic prediction methodologies to enhance the accuracy of associated genetic transmitting abilities in Holstein cows. The objective of this study was to identify and validate SNPs associated with fertility traits impacting early embryo mortality.

**Methods:** The mRNA sequencing data from day 16 normal (*n* = 9) and embryo mortality (*n* = 6) conceptuses from lactating multiparous Holstein cows were used to detect SNPs. The selection of specific genes with SNPs as preliminary candidates was based on associations with reproductive and fertility traits. Validation of candidate SNPs and genotype-to-phenotype analyses were conducted in a separate cohort of lactating primiparous Holstein cows (*n* = 500). After genotyping, candidate SNPs were filtered using a quality control pipeline via PLINK software. Continuous numeric and binary models from reproductive traits were evaluated using the mixed procedure for a generalized linear model-one way ANOVA or logistic regression, respectively.

**Results:** Sixty-nine candidate SNPs were initially identified, but only 23 passed quality control procedures. Ultimately, the study incorporated 466 observations for statistical analysis after excluding animals with missing genotypes or phenotypes. Significant (*p* <0.05) associations with fertility traits were identified in seven of the 23 SNPs: DSC2 (cows with the A allele were older at first calving); SREBF1 and UBD (cows with the T or G alleles took longer to conceive); DECR1 and FASN (cows with the C allele were less likely to become pregnant at first artificial insemination); SREBF1 and BOLA-DMB (cows with the T allele were less likely to be pregnant at 150 days in milk). It was also determined that two candidate SNPs within the DSC2 gene were tag SNPs. Only DSC2 SNPs had an important allele substitution effect in cows with the G allele, which had a decreased age at first calving by 10 days.

**Discussion:** Candidate SNPs found in this study could be used to develop genetic selection tools to improve fertility traits in dairy production systems.

## 1 Introduction

Until 2005, Holstein cows were under intense genetic selection for milk yield, which inadvertently led to a decline in fertility traits (García-Ruiz et al., 2016). This was to be expected given the negative genetic correlations that range from 0.35 to 0.60 for these traits (Pritchard et al., 2013). The advances in the use of genomic information have identified associations with economically relevant traits in cattle and revolutionized animal breeding programs (Matukumalli et al., 2009; Amos et al., 2011; Khatkar et al., 2014). Despite the genetic improvement in Holstein cows, the fertility performance of this breed is still considered suboptimal when compared to other dairy cattle breeds (Martinez-Castillero et al., 2020).

Fertility based on daughter pregnancy rate (DPR) has clearly improved over the last 2 decades (García-Ruiz et al., 2016). Regardless, embryo mortality (EM) is still an issue and was recently reported to be as high as 37% from d17–33 of pregnancy in Holstein cows (Domingues et al., 2024). Pregnancies that result in EM are associated with multiple factors, including inadequate interferon tau (IFNT) signaling by the conceptus (Evans et al., 2012), which plays a key role in establishing and maintaining pregnancy by preventing regression of the corpus luteum (Rizos et al., 2012; Hansen et al., 2017; Moraes et al., 2018). As a result, the production of progesterone from the corpus luteum continues to support conceptus growth and maintain a competent uterine environment for the implanting conceptus (Bazer et al., 1975; Bazer et al., 1997). A challenge with EM pregnancies is that they take place during the pre-implantation period, between days 7 and 16 of pregnancy. Therefore, pregnancy status is unknown until determined at day 32 via ultrasound (Lonergan and Forde, 2014). This hinders resynchronizing non-pregnant cows in a timely manner, limits management of a cow’s reproductive performance, and results in an annual loss of $1.6 billion for the dairy industry in the United States and $1.28 trillion worldwide (Suthar and Shah, 2009; Perkel et al., 2015), further providing a rationale for the selection of Holstein cows with superior fertility traits.

Given that single nucleotide polymorphisms (SNPs) are responsible for 84% of the variation in gene expression, their location in DNA could affect protein structure, production, and function and/or cause a phenotype that could vary due to the reproductive status of an animal (El-Sayed et al., 2006; Beltman et al., 2010; Kommadath et al., 2011; Spencer et al., 2014). Consequently, using SNPs associated with reproductive traits in genomic diagnostic panels may improve genomic estimates of predicted transmitting abilities (Cochran et al., 2013a). Diagnostics using SNP genotyping could further aid in the culling of cattle that are reproductively inferior (Singh et al., 2014).

It was hypothesized that pregnancies with EM are associated with missense SNPs that impair maternal-conceptus communication. We identified 69 candidate SNPs within lactating multiparous Holstein cows that had pregnancies with conceptuses that were either normal (N) or EM and aimed to validate SNP associations in a separate population of lactating primiparous Holstein cows (n = 500).

## 2 Materials and methods

**FIGURE 1:**
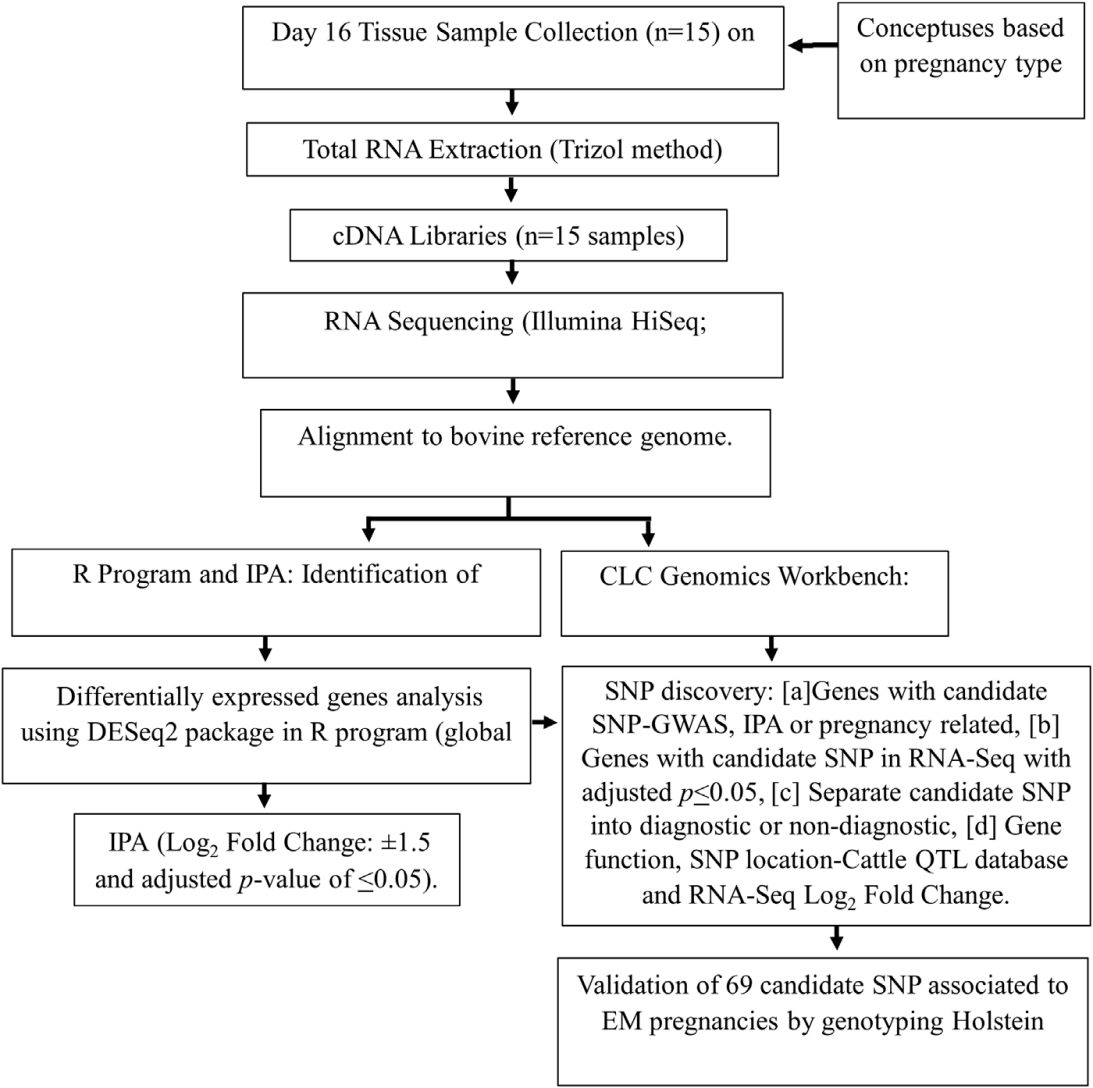
Flowchart of methods used to identify candidate SNPs used in association analysis with reproduction traits in Holstein cows. EM, embryo mortality or N, normal pregnancy: cow’s pregnancy status; IPA, ingenuity pathway analysis.

Figure 1 is a flow chart of the methodology from the initial lactating multiparous Holstein cow population (n = 15). Each cow had a day-16 conceptus collected for the discovery of genes with candidate SNPs that may influence pregnancies to become EM. The candidate SNPs were validated in a separate cohort of lactating primiparous Holstein cows (n = 500) in Colorado, United States.

### 2.1 Animal care

The initial group of fifteen healthy lactating multiparous Holstein cows was located at a dairy farm in North-Eastern Colorado and had conceptuses collected as per approval of Colorado State University’s Institutional Animal Care and Use Committee (IACUC protocol number 17-7539A). An additional 500 healthy lactating primiparous Holsteins from an organic dairy farm in North-Eastern Colorado had blood samples collected in August 2020 (IACUC protocol number 1036). Both groups of cows were fed and milked twice daily, offered water *ad-libitum*, and received a total mixed ration according to the guidelines of the National Research Council (Council, 2001). Cows were also housed in a free-stall barn equipped with sprinklers, fans, and sand-beds.

### 2.2 Estrous cycle synchronization

After 60 days *postpartum*, the initial group of healthy lactating multiparous Holstein cows (n = 15) were subjected to synchronization of the estrous cycle and induced ovulation by the Ovsynch protocol. Cows were exposed to timed artificial insemination (TAI) and the same high-fertile sire’s semen. The day of TAI was considered day 0 for cows in the pregnant group. All were TAI at 16 h after the final intramuscular injection from Ovsynch. If no conceptus was recovered on day 16 after the first TAI, then new cows were assigned to the study.

### 2.3 Recovery and collection of conceptuses

Infusion of flushing media (30 mL of phosphate buffered saline [PBS] and 10 mL of 0.01% of polyvinyl alcohol; Sigma) was performed using a 60 mL plastic syringe (Norm-Ject) connected to a French silicone-coated latex Foley modified catheter (size 22-Bard). The Foley catheter was inserted transcervically into the uterine horn ipsilateral to the corpus luteum. Recovery of conceptuses involved using the same 60 mL syringe and placing the fluid in a sterile petri dish. Each dish was examined under a stereoscope (Stereo Star Zoom, American Optical) at ×7 magnification to find the conceptus. This procedure was repeated up to six times to ensure the recovery of the conceptus, if present. Once the conceptus was located, it was rinsed with sterile PBS and 0.1% (v/v) of polyvinyl alcohol, measured for length (millimeters), and photographed beside a ruler. Pregnancies were re-classified based on conceptus morphology and appearance as N (translucent and elongated [≥100 mm]; n = 9) or undergoing EM (pink, red, opaque and/or restricted elongation [≤60 mm]; n = 6). All conceptuses were snap-frozen with liquid nitrogen and stored at −80°C for RNA extraction.

### 2.4 Conceptus RNA isolation

Total conceptus RNA was extracted using TRIzol reagent (Life Technologies; Carlsbad, California) following the manufacturer’s instructions. The remaining DNA was removed with an RNase-free DNase (Qiagen) and RNAeasy MinElute Cleanup Kit 50 (Qiagen; catalog # 74204). RNA was quantified using a NanoDrop 2000 Spectrophotometer (Thermo Scientific, manufacture: ND2000USCAN). The quality of each RNA sample was determined by dividing the absorbance260 by absorbance280. Values of 2.0 were considered high-quality RNA samples.

### 2.5 Preparation and normalization of RNA-Seq library files

Total conceptus RNA was shipped to be processed for cDNA libraries and sequencing using the Illumina HiSeq (2000) system by Zoetis Inc. Files were single-end sequencing with 75 bp. Sequences were trimmed and aligned to the bovine reference genome ARS-UCD 1.2 (http://bovinegenome.elsiklab.missouri.edu/node/61; (Rosen, 2018).

### 2.6 Identification of differentially expressed genes in RNA sequencing

Using R studio (version 4.1.2; R core team, 2021), RNA-Seq data were exported, organized (dplyr and plyr), and filtered (edgeR) to exclude genes with less than 10 mRNA raw counts. The DESeq2 package (Love et al., 2014) in R used the Benjamini–Hochberg method to control for false discovery by adjusting p-values (Hochberg, 1995). Comparisons (1 × 1) for conceptuses (EM vs. N) were performed using a negative binomial distribution model. Consequently, all differentially expressed genes (DEGs) were identified with adjusted p-values <0.05.

### 2.7 Analysis of RNA-Seq data

The differentially expressed genes (DEGs) from RNA-Seq data were submitted into Ingenuity Pathway Analysis software (IPA; Qiagen) to gain additional biological insight into patterns of gene expression. The core analysis of the data used p < 0.001 and ±2 log2-fold change equivalent to a ±4-fold change in DEGs.

### 2.8 Identification of candidate SNPs within genes

After collecting and processing conceptuses for RNA Seq and aligning their sequences to the bovine reference genome, the Qiagen CLC Genomics Workbench software (version 20.0.1; https://digitalinsights.qiagen.com) was used to identify SNPs within the conceptus RNA-Seq data. The SNP discovery entailed four steps. In the first step, genes with SNPs were selected based on (a) association with reproductive and fertility traits in published genome-wide association studies such as GWAS-SNP (Cochran et al., 2013a; Cochran et al., 2013b; Ortega et al., 2017) or QTLdb (Supplementary Table S3), and (b) DEGs that were identified in the conceptuses of the initial group of Holstein cows using IPA. The second step filtered genes identified in step one by selecting only those genes that were statistically significant in the conceptus RNA-Seq data. The third step separated SNPs into one of two groups: diagnostic (only individuals from one type of pregnancy [N or EM] were used to calculate the frequency of a genotype) or non-diagnostic (individuals from both types of pregnancies [N and EM] were used to calculate the frequency of a genotype). The fourth step consisted of (a) evaluating the function of each gene with SNPs, the region within a gene (i.e., non-synonymous), and the functional classification of SNPs (i.e., missense) via Ensembl Variant Effect Predictor, (b) conducting Sorting Intolerant From Tolerant tool analyses (McLaren et al., 2016) that predicted amino acid substitution affecting protein function by evaluating SNP values (0.0–1.0; closer to zero was predicted to have a significant effect in protein function), (c) verifying if the SNP location within the gene was near (5 centimorgan = 5 million nucleotides) a region/SNP previously associated with a reproductive or fertility trait in the Cattle quantitative trait loci (Cattle QTL; (Hu et al., 2022)) database, and (d) verifying the expression in Log2 fold changes of DEGs with candidate SNPs and their adjusted p-values (<0.05) within the conceptus RNA-Seq data.

### 2.9 Enrollment and collection of records

The SNPs identified in the conceptus RNA-Seq data from the initial lactating multiparous Holstein cows (n = 15) needed to be validated. A separate group of lactating primiparous Holstein cows (n = 500) were randomly enrolled in the study at a dairy milking approximately 9,790 cows. Data of interest were divided into three categories. The first category consisted of health traits that were divided into two sub-categories: reproductive disease (mastitis, metritis, endometriosis, and pyometra) or non-reproductive (lameness, respiratory, digestive, and ketosis). Sub-categories of health traits were recorded on the date of incidence, number of incidences, and type of incidence (0: disease absence or 1: presence of disease) that occurred at or before 60 days of milk (DIM). The second category was comprised of reproductive trait data that were collected after their first calving and up to four artificial inseminations (AIs), which included age (days) at first calving, breeding, conception, and calving date of second pregnancy, season (warm season: June to September or cool season: October to May) that cow was bred and calved, sire identification used for artificial insemination (AI), AI technician identification, number of AI services, pregnancy outcome (pregnant or non-pregnant) and pregnancy loss (0: no pregnancy loss or 1: pregnancy loss; recorded as an abortion after conception). The third category encompassed production traits that were divided into predicted first lactation milk production at 305 DIM, culling of a cow (0: no culling or 1: culling), reason for culling, date of culling, and unit in which cow was housed (location 1 or location 2).

### 2.10 Blood collection

Blood was collected from the tail vein using evacuated tubes containing K2 EDTA (Vacutainer, Becton Dickinson, Franklin Lakes, NJ) while cows were restrained with individual headlocks. Blood samples were placed on ice for transport to the Animal Reproduction and Biotechnology Laboratory facility at Colorado State University. Upon arrival, samples were centrifuged for 30 min at 2,500 rpm to separate blood components. The buffy coat was extracted, placed in a 1.5 mL microcentrifuge tube, suspended up to 1 mL in 1× phosphate-buffered saline, and stored at −20C.

### 2.11 DNA isolation

Genomic DNA was extracted from the buffy coat using the Qiagen DNeasy Blood and Tissue Kit (Cat. No. 69504) according to the manufacturer’s protocol. Sample purity and quality were quantified using a NanoDrop 2000 Spectrophotometer (Thermo Scientific, manufacture: ND2000USCAN) and dividing the absorbance’s wavelength reading, absorbance260 over absorbance280. Values of 2.0 were considered high-quality DNA samples.

### 2.12 Design of custom SNP genotyping panel

A custom SNP genotyping panel for candidate SNPs was designed using the Agena Plex panel (MassARRAY System with 96-well plates) from Neogen^®^ (Lansing, Michigan), which consisted of four steps conducted by Neogen^®^. The first step was providing a list of candidate SNPs with reference identification (RSID) and location of the SNP (150 base pairs up- and down-stream sequence). The second step involved conducting an *in silico* assay design to verify the efficiency and robustness of the assay based on the percentage of SNPs without overlap (less than 150 base pairs) of other SNPs. The SNPs that overlapped were separated into different panels. The third step tested the custom candidate SNP primers for robustness/efficiency by genotyping a subset of the primiparous Holstein cow samples (n = 24). The final step consisted of an optimized panel to genotype the remaining samples (n = 476). Thus, all primiparous Holstein samples (n = 500) were genotyped to validate the candidate SNPs.

### 2.13 Quality control pipeline for candidate SNPs

**FIGURE 2:**
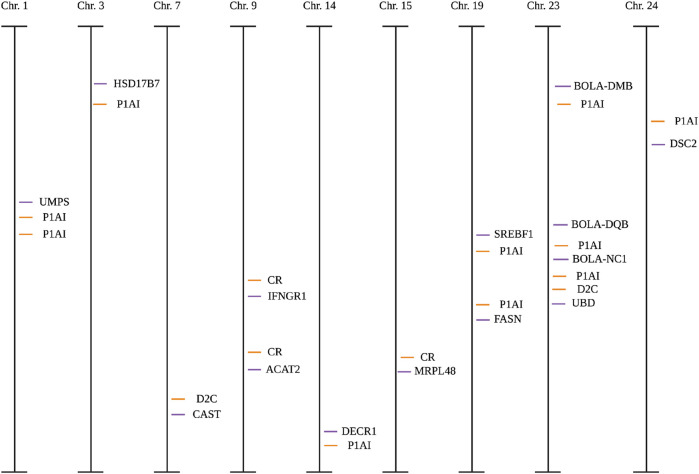
Candidate single-nucleotide polymorphisms (SNPs) and published SNPs associated with quantitative trait loci (QTL, orange lines) within fertility for each chromosome (Chr) in Holstein cows. CR, conception rate; D2C, days to conception; P1AI1, pregnant at first artificial insemination. Orange lines represent published SNPs associated with quantitative trait loci; purple lines represent candidate SNPs. Created with Biorender.com.

Candidate SNPs were filtered after all primiparous Holstein samples (n = 500) were genotyped. The candidate SNPs that were not in minor allele frequency (MAF) of >10% and/or were monomorphic were eliminated from the study. The remaining candidate SNPs were evaluated by creating a five-step quality control pipeline implemented using PLINK software (version 1.07, (Purcell et al., 2007). The first step removed SNPs with 20% missing genotypes. The second step removed individual animals that were not genotyped for 10% of candidate SNPs. The third step removed candidate SNPs not in Hardy–Weinberg equilibrium at a level of significance above 1e−15. The fourth step evaluated the remaining candidate SNPs (Table 1) for linkage disequilibrium via r^2^ and d′ (Supplementary Table S1). The final step identified tag SNPs (Table 2; Figure 2). If more than one tag SNP was identified for a group of SNPs, the tag SNP was selected based on having the highest r^2^ and d′ values (i.e., strongest relationship to the group of SNPs). Additional animals were removed from the study due to missing reproductive (breeding date, calving of cow, and dystocia score) and production trait (predicted milk yield for 305 DIM during cow’s first lactation) data. Thus, the total number of observations used for the statistical models of the study represented 466 cows.

**TABLE 1:** Candidate single-nucleotide polymorphisms (SNPs; *n* = 23) that were non-monomorphic and passed the quality control pipeline using PLINK software in primiparous lactating Holstein cows (*n* = 466).

Gene	RSID^1^	CHRM^2^	SNP Location	Genotype	n	MAF^3^	HWE^4^
*UMPS*	rs110953962	1	69148086	C/T	463	0.29	0.8
*HSD17B7*	rs110828053	3	6635945	C/T	464	0.19	0.5
*CAST*	rs110914810	7	96152634	C/G	466	0.38	1
*IFNGR1*	rs109049057	9	75092093	C/T	466	0.29	0.3
*ACAT2*	rs109967779	9	96041211	A/G	464	0.40	0.6
*DECR1*	rs41580472	14	73708561	C/T	465	0.27	0.9
*MRPL48*		15	53332881	A/G	466	0.48	0.5
*SREBF1*	rs41912290	19	34646676	C/T	465	0.40	0.6
*FASN*	rs41919985	19	50793357	A/G	433	0.29	1.2e^-05^
*BOLA-DMB*	rs109032590	23	7249490	C/T	464	0.30	0.7
*BLA-DQB*	rs109291107	23	25674287	A/G	409	0.20	2e^-13^
*BOLA-NC1*	rs382125666	23	28551269	A/C	401	0.22	2.68e^-06^
*UBD*	rs209518868	23	29119086	A/G	465	0.11	0.007
*UBD*	rs109295136	23	29119334	A/G	400	0.42	3e^-06^
*DSC2*	rs109300814	24	26043125	A/C	463	0.45	3.4e^-12^
*DSC2*	rs210995078	24	26048022	A/G	466	0.45	1
*DSC2*	rs211151260	24	26050992	A/G	466	0.36	0.3
*DSC2*	rs385100256	24	26057277	C/G	466	0.36	0.3
*DSC2*	rs109503725	24	26057282	C/T	466	0.45	1
*DSC2*		24	26060104-5	AA/GT	459	0.44	0.8
*DSC2*	rs109278906	24	26060155	A/T	463	0.44	1
*DSC2*	rs110651429	24	26060157	C/T	464	0.44	0.9
*DSC2*	rs210416248	24	26063437	A/G	466	0.36	0.3

^1^RSID, reference SNP identification; 2CHRM, chromosome; 3MAF, minor allele frequency; 4HWE, Hardy–Weinberg equilibrium.

**TABLE 2:** Candidate single-nucleotide polymorphisms (SNPs; *n* = 8) with a tag SNP using PLINK software in primiparous lactating Holstein cows.

Gene	RSID^1^	CHRM^2^	SNP location	Tag SNP	Tag SNP RSID	Tag SNP location
*DSC2*	rs210995078	24	26048022	*DSC2*	rs109278906	26060155
*DSC2*	rs109503725	24	26057282	*DSC2*	rs109278906	26060155
*DSC2*	rs110651429	24	26060157	*DSC2*	rs109278906	26060155
*DSC2*		24	26060104-5	*DSC2*	rs109278906	26060155
*DSC2*	rs385100256	24	26057277	*DSC2*	rs211151260	26050992
*DSC2*	rs210416248	24	26063437	*DSC2*	rs211151260	26050992
*DSC2*	rs210995078	24	26048022	*DSC2*	rs109278906	26060155

RSID^1^, reference SNP identification; CHRM^2^, chromosome. *DSC2*, desmocollin-2.

### 2.14 Statistical analysis

Genotype-phenotype statistical association models were evaluated for services per conception, age at first calving, days to conception, days to first AI, pregnant at first AI, pregnant at 150 DIM, and pregnancy loss (Table 3). The independent variables included within each model were genotypes, reproductive disease, non-reproductive disease, and housing unit. Each of the reproductive traits used as models was evaluated for mean, standard deviation, minimum, and maximum via the means procedure of SAS (SAS, 2023) (Table 4). Continuous numeric traits (services per conception, age at first calving, days to conception, and days to first AI) and binary models (i.e., 1: event occurred or 0: event did not occur; pregnant at first AI, pregnant at 150 DIM, and pregnancy loss) were evaluated for statistical significance with and without a single genotype term from candidate SNPs by using the generalized linear model-one way ANOVA or logistic regression, respectively. The r^2^ was calculated using McFadden’s r squared, which is calculated by the proc glm command and evaluated with and without the genotype term to demonstrate the amount of variation explained by the model’s inputs. Only statistically significant models with a single genotype term from candidate SNPs were evaluated using the means separation test within LSMEANS, which included the Bonferroni adjustment for p-values to minimize false discovery error from the mixed procedure (Weir, 2001). Interactions between genotype terms and other fixed effects were also evaluated but only among those sharing the same reproductive trait model. Furthermore, we evaluated the effect of genotype additive and dominance allele effects on candidate SNPs (Luna-Nevarez et al., 2011). Linear and quadratic contrasts were executed to confirm or reject such additive or dominant allele effects. Statistical procedures were conducted in SAS OnDemand for academic software, and statistical significance was defined as P ≤ 0.05.

**TABLE 3:** Basic model, and qualitative and quantitative covariate class variables used to predict the categorical traits.

Statistical model
Y_ijklm_ = μ + genotype_i_ + age at first calving_j_ + reproductive disease_k_ + non-reproductive disease_l_ + housing unit_m_ + e_ijklm_

^c^ ^a^Y_ijkmqr_ = qualitative trait (services per conception_b_, age at first calving_b_, days to conception_b_, days to first AI_b_, pregnant at first AI_c_, pregnant at 150 DIM_c_, or pregnancy loss_c_) for the *r*th cow in the (i, j, k, l, m)th cell; μ, general mean for the reproductive trait; genotype_i_, fixed effect of genotypes; age at first calving_j_, covariate effect of the age at first calving; reproductive disease_k_, fixed effect of the reproductive disease; non-reproductive disease_l_ , fixed effect of the non-reproductive disease; housing unit_m_, fixed effect of the housing unit; e_ijklm_, random error term. ^b^Non-binary models that were evaluated with GLM one-way ANOVA.^C^Binary models that were evaluated with logistic regression.

**TABLE 4:** Summary statistics for study populations, including MEANS, GLM^a^, and Logistic^b^ for conception, age at calving, and pregnancy in primiparous lactating Holstein cows.

Trait	n	Mean +SD^1^	Min^2^	Max^3^	p-value	X^2^ test p-value
Services per conception^a^	466	3.8 ± 2.8	1	13	0.001*	
Age at first calving, days^a^	466	751.2 ± 62.1	560	993	0.1	
Days to conception^a^	417	159.2 ± 102.5	45	579	0.0105*	
Days to first AI^a^	466	75.7 ± 32.4	37	266	<0.0001*	
Pregnant at first AI^b^	466	0.2 ± 0.4	0	1		0.1
Pregnant at 150 DIM^b^	466	0.5 ± 0.5	0	1		0.008*
Pregnancy loss^b^	466	0.07 ± 0.2	0	2		0.6

^a^ Non-binary models that were evaluated with GLM one-way ANOVA.

^b^ Binary models that were evaluated with logistic regression. *Models that were statistically significant (P≤ 0.05) without genotype term. SD1 = standard deviation; Min2 = minimum; Max3 = maximum. AI, artificial insemination; DIM, days in milk.

## 3 Results

### 3.1 Quality control pipeline for candidate SNP

Sixty-nine candidate SNPs were discovered within the RNA-Seq data of EM compared to N conceptuses from a multiparous population of Holstein cows (n = 15). All candidate SNPs were validated in a separate population of primiparous Holstein cows (n = 500). Only 30 of the 69 candidate SNPs were in MAF and were non-monomorphic. The remaining candidate SNPs were evaluated through a five-step quality control pipeline using PLINK software. The final number of candidate SNPs and individual animals for this study were 23 and 466, respectively (Table 1). Eight of the 23 candidate SNPs were in linkage disequilibrium via r^2^ and d′ (Supplementary Table S1), and two within the DSC2 gene were identified as tag SNPs (Table 2; Supplementary Figure S1).

### 3.2 Statistically associated SNPs

**FIGURE 3:**
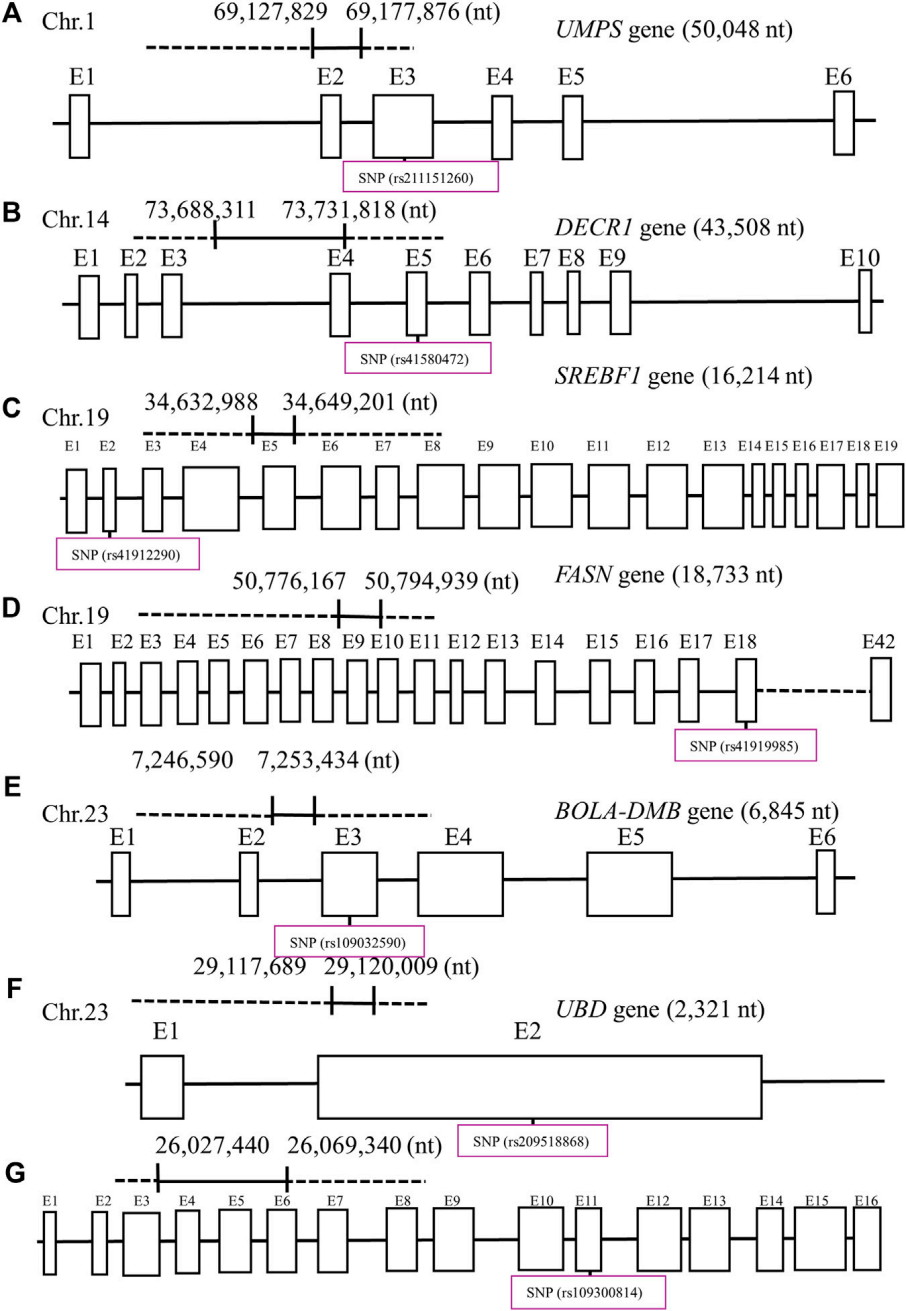
Seven potential functional single-nucleotide polymorphisms (SNPs) within exons (E) and nucleotide (nt) position in each gene: **(A)** uridine monophosphate synthetase (*UMPS*), **(B)** 2,4-dienoyl-CoA reductase 1 (*DECR1*), **(C)** sterol regulatory element-binding transcription factor 1 (*SREBF1*), **(D)** fatty acid synthetase (*FASN*), **(E)** major histocompatibility complex, class II, DM beta (*BOLA-DMB*), **(F)** ubiquitin d (*UBD*), and **(G)** desmocollin-2 (*DSC2*) in their designated chromosome (Chr) for Holstein cows. Created with Biorender.com.

All candidate SNPs were in proximity (<5 centimorgans) of at least one SNP associated with fertility traits reported in the Cattle QTL database (Figure 2). All models were evaluated for reproductive traits with single genotype terms and nine instances that differed (p < 0.05; Table 6; Supplementary Table S2; Figure 3). The R^2^ of a model was greater when the genotype term was significant (p < 0.05; Table 5).

**TABLE 5:** Summary of R^2^ of the model without and with potential functional candidate single-nucleotide polymorphisms (SNPs; n = 7) in primiparous lactating Holstein cows.

Trait	Gene	RSID^1^	n	R^2^ without SNP	*p*-value	n	R^2^ with SNP	*p*-value
Age at first calving, days	*DSC2*	rs211151260	466	0.01	0.1	466	0.02	0.04*
Days to conception	*SREBF1*	rs41912290	417	0.03	0.01*	416	0.04	0.003*
	*UBD*	rs209518868	417	0.03	0.01*	416	0.05	0.001*
Days to first AI	*SREBF1*	rs41912290	466	0.06	<0.0001*	465	0.08	<0.0001*
	*UMPS*	rs110953962	466	0.06	<0.0001*	463	0.07	<0.0001*
Pregnant at first AI	*DECR1*	rs41580472	466	0.01	0.1042	465	0.03	0.02*
	*FASN*	rs41919985	466	0.01	0.1042	433	0.03	0.01*
Pregnant at 150 DIM	*SREBF1*	rs41912290	466	0.02	0.008*	465	0.04	0.001*
	*BOLA-DMB*	rs109032590	466	0.02	0.008*	464	0.04	0.002*

*Candidate SNPs that were statistically significant (p < 0.05) within models. RSID^1^, reference SNP identification; AI, artificial insemination; DIM, days in milk. DSC2, desmocollin-2; SREBF1, sterol regulatory element-binding transcription factor 1; UBD, ubiquitin d; UMPS, uridine monophosphate synthetase; DECR1, 2, 4-dienoyl-CoA reductase 1; FASN, fatty acid synthetase; BOLA-DMB, major histocompatibility complex, class II, DM beta.

Cows that were older at first calving were associated with carrying an A allele in the desmocollin-2 (DSC2; rs211151260; Table 6) SNP. Meanwhile, cows that carried a T allele e in sterol regulatory element-binding transcription factor 1 (SREBF1; rs41912290) or a G allele in ubiquitin d (UBD; rs209518868; Table 6) were associated with having longer intervals to become pregnant after calving. Cows might experience such intervals due to reproductive diseases impacting the health of the animal, the unit in which cows were housed, or their interactions, but that was not the case for both the SREBF1 and UBD SNPs. Moreover, no interactions were found between the SREBF1 and UBD SNPs. For the SNPs of uridine monophosphate synthetase (UMPS; rs110953962) and SREBF1 (rs41912290), cows that carried a T allele were associated with being AI’d later than other cows (Table 6). For both models, age and reproductive diseases were significant (p < 0.001). Age and disease interactions between UMPS or SREBF1 SNPs were not different in days to first AI. Cows associated with being less likely to become pregnant at first AI had a C allele in 2, 4-dienoyl-CoA reductase 1 (DECR1; rs41580472) or an A allele in fatty acid synthetase (FASN; rs41919985; Table 6). No differences were observed for other variables except for the genotype term of the DECR1 and FASN SNPs (p < 0.05). In addition, interactions (p < 0.05) were found between the DECR1 and FASN SNPs. Cows were less likely to become pregnant before or at 150 DIM with a T allele in SREBF1 (rs41912290) or in the major histocompatibility complex, class II, DM beta (BOLA-DMB; rs109032590; Table 6) when compared to those that had at least one C allele. No differences were found for variables except for the genotype terms of SREBF1 and BOLA-DMB SNPs (p < 0.05). Interactions (p < 0.01) were also found between the SREBF1 and BOLA-DMB SNPs in pregnant cows at 150 DIM.

**TABLE 6:** Least square means ± standard error for fertility traits among genotypes within genes with candidate single-nucleotide polymorphisms (SNPs) in primiparous lactating Holstein cows.

Trait	Gene	RSID^1^	n	Allele combination		
Age at first calving, days	*DSC2*	rs211151260	466	AA: 761.9 ± 8.7	AG: 757.8 ± 5.7	GG: 744.1 ± 5.7
Days to conception	*SREBF1*	rs41912290	416	CC: 176.3 ± 10.9	CT: 162.5 ± 10.09	TT: 197.7 ± 14.04
	*UBD*	rs209518868		AA: 173.1 ± 9.008	AG: 175.7 ± 12.5	GG: 481.83 ± 101.2
Days to first AI	*SREBF1*	rs41912290	465	CC: 80.08 ± 3.1	CT: 80.5 ± 2.8	TT: 91.4 ± 3.9
	*UMPS*	rs110953962	463	CC: 83.01 ± 2.7	CT: 79.2 ± 3.04	TT: 93.03 ± 5.3
Pregnant at first AI	*DECR1*	rs41580472	465	CC: 0.2 ± 0.03	CT: 0.2 ± 0.04	TT: 0.4 ± 0.07
	*FASN*	rs41919985	433	AA: 0.01 ± 0.09	AG: 0.2 ± 0.03	GG: 0.1 ± 0.04
Pregnant at 150 DIM	*SREBF1*	rs41912290	465	CC: 0.4 ± 0.04	CT: 0.4 ± 0.04	TT: 0.3 ± 0.06
	*BOLA-DMB*	rs109032590	464	CC: 0.4 ± 0.04	CT: 0.5 ± 0.04	TT: 0.3 ± 0.08

*Candidate SNPs that were statistically significant (p < 0.05) within models. RSID^1^, reference SNP identification; AI, artificial insemination; DIM, days in milk. DSC2, desmocollin-2; SREBF1, sterol regulatory element-binding transcription factor 1; UBD, ubiquitin d; UMPS, uridine monophosphate synthetase; DECR1, 2, 4-dienoyl-CoA reductase 1; FASN, fatty acid synthetase; BOLA-DMB, major histocompatibility complex, class II, DM beta.

All of the potential functional SNPs were non-synonymous and classified as missense. Three of the seven potential functional SNPs were predicted to influence the protein function of the gene (p < 0.05; Table 7). All seven potential functional SNPs had an additive effect (p < 0.05) due to a linear trend observed when the genotype term was a fixed effect within the model (Table 8). Conversely, the DSC2 and SREBF1 SNPs had allele substitution effects (p < 0.05; Table 8), while only the DECR1 SNP tended (p < 0.10; Table 8) to yield differing levels among genotypes. Cows with allele G in DSC2 (p < 0.05) had a 10-day decreased age at first calving (Table 8). Meanwhile, cows with a C allele in SREBF1 impaired the trait level and had a 6% decreased (p < 0.05) probability of becoming pregnant at 150 DIM (Table 8).

**TABLE 7:** Variant effect predictor analysis for SNPs within genes with candidate single-nucleotide polymorphisms (SNPs) in primiparous lactating Holstein cows.

Coding region	Type of SNP	Gene	RSID^1^	Exon	Codon change	Amino acid change	SIFT
Non-synonymous	Missense	*DSC2*	rs211151260	11	**G**AA/**A**AA	**E**/**K**	0.01*
		*SREBF1*	rs41912290	2	C**C**T/C**T**T	**P**/**L**	0.03*
		*UBD*	rs209518868	2	A**A**G/A**G**G	**K**/**R**	0.4
		*UMPS*	rs211151260	3	**C**GT/**T**GT	**R**/**C**	0.01*
		*DECR1*	rs41580472	5	**A**TA/**G**TA	**I**/**V**	0.06
		*FASN*	rs41919985	18	**A**CC/**G**CC	**T**/**A**	0.7
		*BOLA-DMB*	rs109032590	3	**A**TC/**G**TC	**I**/**V**	0.5

*Candidate SNPs that were statistically significant (p < 0.05) within models. RSID^1^, reference SNP identification. DSC2, desmocollin-2; SREBF1, sterol regulatory element-binding transcription factor 1; UBD, ubiquitin d; UMPS, uridine monophosphate synthetase; DECR1, 2, 4-dienoyl-CoA reductase 1; FASN, fatty acid synthetase; BOLA-DMB, major histocompatibility complex, class II, DM beta.

**TABLE 8:** Allele substitution estimates and fixed effect estimates of additive and dominance of the genes with candidate single-nucleotide polymorphisms (SNPs) in primiparous lactating Holstein cows.

Trait	Gene	n	Allele substitution effects			Fixed effects		
			p-value^a^	Estimate^b^	SE^1^	p-value^c^	Additive effect^d^	Dominant effect^e^
A1C, days	*DSC2*	466	0.01*	−10.09	4.1	0.03*	8.9	748.9
D2C	*SREBF1*	416	0.4	5.8	7.2	0.04	10.7	151.8
	*UBD*	416	0.4	9.7	11.7	0.01	308.7	132.9
D21AI	*SREBF1*	465	0.02*	4.7	2.08	0.01*	5.6	74.8
	*UMPS*	463	0.5	1.2	2.2	0.04	5.01	74.2
P1AI	*DECR1*	465	0.05	0.06	0.03	0.01	0.1	0.1
	*FASN*	433	0.7	−0.01	0.03	0.01	0.08	0.1
P150 DIM	*SREBF1*	465	0.04*	−0.06	0.03	0.01*	0.08	0.1
	*BOLA-DMB*	464	0.3	0.03	0.03	0.04	−0.07	0.07

^a^p-values obtained from allele substitution analysis in SAS, which included the term genotype as a covariate. ^b^Estimates of the effect expressed in units of the traits. ^c^Bonferroni corrected p-values for fixed effects were obtained from the substitution of favorable allele analysis, which included the genotype term as a fixed effect. ^d^Additive effect was estimated as the difference between the two homozygous genes means divided by 2. ^e^Dominant effect was calculated as the deviation of the heterozygous genes from the mean of the two homozygous. *Candidate SNPs that were statistically significant (p< 0.05) within models. SE^1^ = standard error. AIC, age at first calving; D2C, days to conception; D21AI, days to first artificial insemination; P1AI, pregnant at first artificial insemination; P150DIM, pregnant at 150 days in milk. DSC2, desmocollin-2; SREBF1, sterol regulatory element-binding transcription factor 1; UBD, ubiquitin d; UMPS, uridine monophosphate synthetase; DECR1, 2, 4-dienoyl-CoA reductase 1; FASN, fatty acid synthetase; BOLA-DMB, major histocompatibility complex, class II, DM beta.

## 4 Discussion

### 4.1 Functional SNPs

The discovery of functional SNPs could contribute to improving breeding value estimation and genetic improvement of Holstein cows despite the low heritability of fertility traits. Improvement of these strategies could also have a direct impact on the economic efficacy and culling practices of the dairy industry (Pryce et al., 1997; Inchaisri et al., 2010). Among the seven reproductive traits (services per conception, age at first calving, days to conception, days to first AI, pregnant at first AI, pregnant at 150 DIM, and pregnancy loss) that were evaluated in this study, five SNPs may be expected to change amino acids and, based on the substitution effects, could be predicted to be damaging to protein function. Additional biochemical research into these proteins is needed to fully understand how these SNPs influence the variance of these traits.

Identification of at least one QTL associated with fertility traits was found in proximity to this study’s candidate SNPs. Others have shown that QTL has an association with causing an effect over nearby SNPs that control similar traits (Khatkar et al., 2004; Daetwyler et al., 2008). When conducting genotype-to-phenotype association studies, SNPs that are associated with QTL(s) may be helpful when implemented into breeding programs to improve the prediction of traits of low heritability (Spelman and Garrick, 1997; Van Tassell et al., 1999; Abdel-Azim and Freeman, 2002).

All candidate SNPs that were statistically significant (p < 0.05) within the models demonstrated a larger R^2^ value (Table 5). The optimum age of cows at first calving averages between 23 and 24 months because of the cost of rearing replacement heifers in dairy herds (Heinrichs, 1993; Hoffman, 1997; Gabler et al., 2000; Tozer and Heinrichs, 2001; Boulton AC, 2015). In the context of this study’s DSC2 SNP, cows with the G allele were older during their first calving. The DSC2 gene is known for its role in cell junction and adhesion (Lewin et al., 2022); therefore, it has been studied for its potential role in the development and function of bovine embryos via compaction and cavitation (Wrenzycki et al., 2001). The DSC2 SNP has only been studied in an Iranian *Bos taurus* breed, and no associations to age at first calving were identified in Holstein cows. Only a different SNP within DSC2 has been associated with fertility traits of daughter pregnancy rate in Holstein cows (Cochran et al., 2013a).

The interval of conception after calving is often used as a parameter to determine the reproductive performance of dairy cows (Harman et al., 1996), and, ideally, conception takes place prior to 85 days post calving (Grimard et al., 2006; Tillard et al., 2008). However, the interval for average days of conception after calving is highly dependent on diverse factors such as season (i.e., summer), peripartum disorders (i.e., metritis and endometritis), and cow management (Grimard et al., 2006; Tillard et al., 2008; Siddiqui et al., 2013). Minimizing the number of days that a cow is not pregnant (open) is crucial to decreasing costs (i.e., food, labor, breeding) through increasing culling rates as well as the number of calves and longevity of the animal (González-Recio et al., 2004; Inchaisri et al., 2010; Cabrera, 2014). Within our data, we identified the T allele in SREBF1 and the G allele in UMD SNP as being associated with cows having longer intervals of conception after calving. The SREBF1 gene function has been documented as an important regulator for the transcription of genes that synthesize milk fat and its secretion in the mammary epithelial cells of dairy cows (Harvatine and Bauman, 2006; Ma and Corl, 2012; Oppi-Williams et al., 2013; Li et al., 2014). The SREBF1 SNP discussed in this study has been submitted to the ENSEMBL SNP database and is associated with fatty acid indexes that influence milk fat and protein percentage in Holstein cows (Rincon et al., 2012; Cochran et al., 2013a). Despite this, associations for the SREBF1 SNP and its impact on days to conception have not been reported in Holstein cows. The UMD gene has general roles in DNA replication, recombination and repair, and cell development, assembly, maintenance, survival, and death (Salehi et al., 2016). Other discovered SNPs within UBD have mainly been reported to regulate responses by the immune system (Russell et al., 2012; Thompson-Crispi et al., 2014). Although our study’s UBD SNP has been reported in fourteen different cow and bull populations, no association with days to conception has been made in Holstein cows.

The average days to first AI measure was reported to range from 67 to 84 (Esslemont, 1992; Darwash et al., 1997; Royal et al., 2000; Waldmann et al., 2001; Yusuf et al., 2011), favoring an extension of DIM due to higher conception rates being obtained in later rather than earlier stages of lactation (Dohoo, 1983; Royal et al., 2000; Quintela Arias et al., 2004; Bouchard E, 2008). Our data revealed that cows that carried a T allele for UMPS or SREBF1 SNPs were associated with being AI’d 10 days later than cows who carried at least 1 C allele (Table 7). Studies on the UMPS gene have suggested that it has a role in the synthesis of nucleotides in both DNA and RNA (Healey and Shanks, 1987). Deficiency (inactivation) of UMPs, also known as DUMPs, is an inherited recessive disorder that causes arrested growth and development of pregnancies, which leads to EM in Holstein cows (Shanks and Robinson, 1990; Shanks and Greiner, 1992; Robinson et al., 1993; Kuhn and Shanks, 1994). Furthermore, carriers of DUMPs have been found to remain non-pregnant for longer periods of time (Čítek and Barbora, 2004). Prevention of this condition consists of early detection of defect carriers by screening herds routinely through haplotype tests (VĂTĂŞEscu-Balcan et al., 2006). The UMP SNPs identified herein have only been reported within an Iranian *B. taurus* breed, but no associations have been made to days to first AI in Holstein cows. Likewise, no associations of the SREBF1 SNP identified in this study were made in other studies with days to first AI in Holstein cows.

Pregnancy at first AI, which is similar to the trait of days to conception, depends on the time of year, voluntary waiting period, reproductive/peripartum disorders, and reproductive management (Grimard et al., 2006; Tillard et al., 2008; Siddiqui et al., 2013). It has also been proven that dairy cows serviced more than once have reduced pregnancy rates at first AI (77.3% vs. 35.7%) and a 4.5% decreased probability of becoming pregnant for each previously unsuccessful service (Barrett, 1948; Sprott et al., 1998). Our data suggested that the probability of cows becoming pregnant at first AI was lower when they had a C allele in the DECR1 SNP or an A allele in the FASN SNP. The DECR1 gene acts as a mitochondrial enzyme involved in beta-oxidation that regulates the rate of fatty acid metabolism, which contributes to energy production (Kunau and Dommes, 1978; Wathes et al., 2012). The SNP within DECR1 was associated with an effect on lipid metabolism, milk production, back fat thickness, days to first service, and calving interval in beef cattle (Marques et al., 2009; Clempson et al., 2012). Boussaha et al. (2015) submitted the DECR1 SNP within a GWAS data set, but no other publications have associated this submission with pregnancy at first AI in Holstein cows.

The FASN gene role has been associated with catalyzing the production of long-chain fatty acids and has been evaluated as a candidate for improving fat levels in both milk and muscle of cattle (Roy et al., 2006; Schennink et al., 2009; Matsumoto et al., 2012; Li et al., 2016). The SNP within FASN was found to be associated with lactation traits, composition of fatty acids within milk, and reconstitution of body reserves during gestation (Matsumoto et al., 2012; Elis et al., 2013; Mauric et al., 2019) but not with pregnancy at first AI.

Pregnancy that takes place before or at 150 DIM is contingent on reproductive diseases, detection of estrus, voluntary waiting period, and season (Grimard et al., 2006; Tillard et al., 2008; Siddiqui et al., 2013). Timing when pregnancy occurs in dairy cows is critical in sustaining profitability in the industry (Giordano et al., 2011). Both SREBF1 and BOLA-DMB were found to have SNPs in our data associated with a lower probability of becoming pregnant before or at 150 DIM. In the case of the SREBF1 SNP, no associations with pregnancy at 150 DIM have been made. The BOLA-DMB gene is part of the bovine immune system’s major histocompatibility complex in the class IIb region, and it is responsible for aiding with the loading of peptides in antigen-presenting cells (Pathak et al., 2001; La Rocca et al., 2014). Interestingly, the regulation of BOLA-DMB is different in the endometrium of cows that are pregnant compared to non-pregnant cows (Cerri et al., 2012). Associations for this study’s BOLA-DMB SNP were made for daughter pregnancy rate, cow conception rate, heifer pregnancy rate, and milk yield (Cochran et al., 2013a; Ortega et al., 2016; Ortega et al., 2017; Ortega, 2018) but not specifically to pregnancy at 150 DIM.

For allele substitution and additive effects, cows with the beneficial G allele within DSC2 had improved age of first calving, which decreased by 10 days (Table 8). Eastham et al. (2018) reported that cows that were younger during their first calving had an association with udder health, increased longevity, milk yield, improved reproductive performance, and increased probability of calving for a second time. Cows’ age at first AI was demonstrated to be dependent on the management of the herd and was unique to each individual dairy (Esslemont, 1992; Darwash et al., 1997; Royal et al., 2000; Waldmann et al., 2001; Yusuf et al., 2011). Moreover, it has also been shown that cows had higher conception rates when AI occurs at later stages of lactation (Dohoo, 1983; Royal et al., 2000; Quintela Arias et al., 2004; Bouchard E, 2008). Pregnancy occurring before or at 150 DIM is of importance to sustain profitability within the industry, and it optimally occurs between 90 and 130 DIM (Giordano et al., 2011). Thus, the candidate SNPs associated with pregnant at 150 DIM may somewhat be unique to the reproductive management practices of an organic farm, causing longer times for each lactation cycle (Sorge et al., 2016).

This study validated the SNPs identified within the conceptuses (EM and N) of the initial herd of Holstein cows by using an independent herd through genotype-to-phenotype associations. Validation of candidate SNPs using blood samples to evaluate gene expression of reproductive phenotypes has proven useful for genetic panels/evaluations performed in cattle, such as expected progeny differences, predictive transmissibility, or genomic breeding values (i.e., Clarifide from Zoetis). Therefore, developing breeding values that encompass fertility traits is of great value when predicting the expected fertility of replacement Holstein heifers and cows that should remain in the herd (Weigel, 2006; García-Ruiz et al., 2016).

## 5 Conclusion

Genotype-to-phenotype analysis of 69 candidate SNPs suggested that seven SNPs were associated with fertility traits that are of economic importance within Holstein cows that are reproductively inferior. Additional research should be conducted to determine the utility of these candidate SNPs in genome-enhanced predictive transmissibility abilities estimations and (or) commercial genotyping tools for early life sorting of heifers. Thus, future use of said SNPs might be useful for culling decisions upon validation upon validation if they are predictive.

## Data Availability

The datasets presented in this study can be found in online repositories. The names of the repository/repositories and accession number(s) can be found at: https://www.ncbi.nlm.nih.gov/geo/, GSE233492.

## Abbreviations

AI, artificial insemination; DEG, differentially expressed gene; DIM, days in milk; EM, embryo mortality; N, normal conceptus; MAF, minor allele frequency; RNA-Seq, RNA sequencing; SNP, single-nucleotide polymorphism; QTL, quantitative trait loci.
