# Supplementary material for: Identification of candidate SNPs associated with embryo mortality and fertility traits in lactating Holstein cows
Source: Front Genet. 2024 Aug 9;15:1409335. doi: 10.3389/fgene.2024.1409335 (PMC11341358; doi:10.3389/fgene.2024.1409335)
Supplement: Supplementary file 1 [file Table1.DOCX]

| **Supplementary table 1.** Candidate single nucleotide polymorphism (SNP; n= 12 out of 23) that were in linkage disequilibrium (r2 and d’) using PLINK software in primiparous lactating Holstein cows (n=428). | | | | | | | | |  |
| --- | --- | --- | --- | --- | --- | --- | --- | --- | --- |
| **SNP A SNP A SNP B SNP B**  **Gene RSID^1^  CHRM^2^ location SNP B RSID location r^2^ d’** | | | | | | | | |  |
| *BOLA-NC1* | rs382125666 | 23 | 69148086 | *UBD* | rs209518868 | 29119086 | 0.00165162 | 0.0737591 | |
| *BOLA-NC1* | rs382125666 | 23 | 69148086 | *UBD* | rs109295136 | 29119334 | 0.0943097 | 0.477845 | |
| *UBD* | rs209518868 | 23 | 29119086 | *UBD* | rs109295136 | 29119334 | 0.104576 | 0.908803 | |
| *DSC2* | rs109300814 | 24 | 26043125 | *DSC2* | rs210995078 | 26048022 | 0.705424 | 1 | |
| *DSC2* | rs109300814 | 24 | 26043125 | *DSC2* | rs211151260 | 26050992 | 0.665203 | 1 | |
| *DSC2* | rs109300814 | 24 | 26043125 | *DSC2* | rs385100256 | 26057277 | 0.665203 | 1 | |
| *DSC2* | rs109300814 | 24 | 26043125 | *DSC2* | rs109503725 | 26057282 | 0.693562 | 1 | |
| *DSC2* | rs109300814 | 24 | 26043125 | *DSC2* | . | 26060104-5 | 0.677046 | 0.97805 | |
| *DSC2* | rs109300814 | 24 | 26043125 | *DSC2* | rs109278906 | 26060155 | 0.687309 | 0.994494 | |
| *DSC2* | rs109300814 | 24 | 26043125 | *DSC2* | rs110651429 | 26060157 | 0.695425 | 1 | |
| *DSC2* | rs109300814 | 24 | 26043125 | *DSC2* | rs210416248 | 26063437 | 0.653245 | 1 | |
| *DSC2* | rs210995078 | 24 | 26048022 | *DSC2* | rs211151260 | 26050992 | 0.4594 | 1 | |
| *DSC2* | rs210995078 | 24 | 26048022 | *DSC2* | rs385100256 | 26057277 | 0.4594 | 1 | |
| *DSC2* | rs210995078 | 24 | 26048022 | *DSC2* | rs109503725 | 26057282 | 0.983269 | 1 | |
| *DSC2* | rs210995078 | 24 | 26048022 | *DSC2* | . | 26060104-5 | 0.965927 | 0.982816 | |
| *DSC2* | rs210995078 | 24 | 26048022 | *DSC2* | rs109278906 | 26060155 | 0.978915 | 0.995715 | |
| *DSC2* | rs210995078 | 24 | 26048022 | *DSC2* | rs110651429 | 26060157 | 0.983198 | 1 | |
| *DSC2* | rs210995078 | 24 | 26048022 | *DSC2* | rs210416248 | 26063437 | 0.45117 | 1 | |
| *DSC2* | rs211151260 | 24 | 26050992 | *DSC2* | rs385100256 | 26057277 | 1 | 1 | |
| *DSC2* | rs211151260 | 24 | 26050992 | *DSC2* | rs109503725 | 26057282 | 0.451713 | 1 | |
| *DSC2* | rs211151260 | 24 | 26050992 | *DSC2* | . | 26060104-5 | 0.436002 | 0.975585 | |
| *DSC2* | rs211151260 | 24 | 26050992 | *DSC2* | rs109278906 | 26060155 | 0.44366 | 0.991819 | |
| *DSC2* | rs211151260 | 24 | 26050992 | *DSC2* | rs110651429 | 26060157 | 0.452195 | 1 | |
| *DSC2* | rs211151260 | 24 | 26050992 | *DSC2* | rs210416248 | 26063437 | 0.982087 | 1 | |
| *DSC2* | rs385100256 | 24 | 26057277 | *DSC2* | rs109503725 | 26057282 | 0.451713 | 1 | |
| *DSC2* | rs385100256 | 24 | 26057277 | *DSC2* | . | 26060104-5 | 0.436002 | 0.975585 | |
| *DSC2* | rs385100256 | 24 | 26057277 | *DSC2* | rs109278906 | 26060155 | 0.44366 | 0.991819 | |
| *DSC2* | rs385100256 | 24 | 26057277 | *DSC2* | rs110651429 | 26060157 | 0.452195 | 1 | |
| *DSC2* | rs385100256 | 24 | 26057277 | *DSC2* | rs210416248 | 26063437 | 0.982087 | 1 | |
| *DSC2* | rs109503725 | 24 | 26057282 | *DSC2* | . | 26060104-5 | 0.983017 | 1 | |
| *DSC2* | rs109503725 | 24 | 26057282 | *DSC2* | rs109278906 | 26060155 | 0.995765 | 1 | |
| *DSC2* | rs109503725 | 24 | 26057282 | *DSC2* | rs110651429 | 26060157 | 1 | 1 | |
| *DSC2* | rs109503725 | 24 | 26057282 | *DSC2* | rs210416248 | 26063437 | 0.443622 | 1 | |
| *DSC2* | . | 24 | 26060104-5 | *DSC2* | rs109278906 | 26060155 | 0.995702 | 1 | |
| *DSC2* | . | 24 | 26060104-5 | *DSC2* | rs110651429 | 26060157 | 0.987178 | 1 | |
| *DSC2* | . | 24 | 26060104-5 | *DSC2* | rs210416248 | 26063437 | 0.449772 | 1 | |
| *DSC2* | rs109278906 | 24 | 26060155 | *DSC2* | rs110651429 | 26060157 | 1 | 1 | |
| *DSC2* | rs109278906 | 24 | 26060155 | *DSC2* | rs210416248 | 26063437 | 0.446917 | 1 | |
| *DSC2* | rs110651429 | 24 | 26060157 | *DSC2* | rs210416248 | 26063437 | 0.446088 | 1 | |
| RSID^1^=reference SNP identification; CHRM^2^=chromosome. | | | | | | | | | |
