# Supplementary figures and images for: Identification of candidate SNPs associated with embryo mortality and fertility traits in lactating Holstein cows
Source: Front Genet. 2024 Aug 9;15:1409335. doi: 10.3389/fgene.2024.1409335 (PMC11341358; doi:10.3389/fgene.2024.1409335)

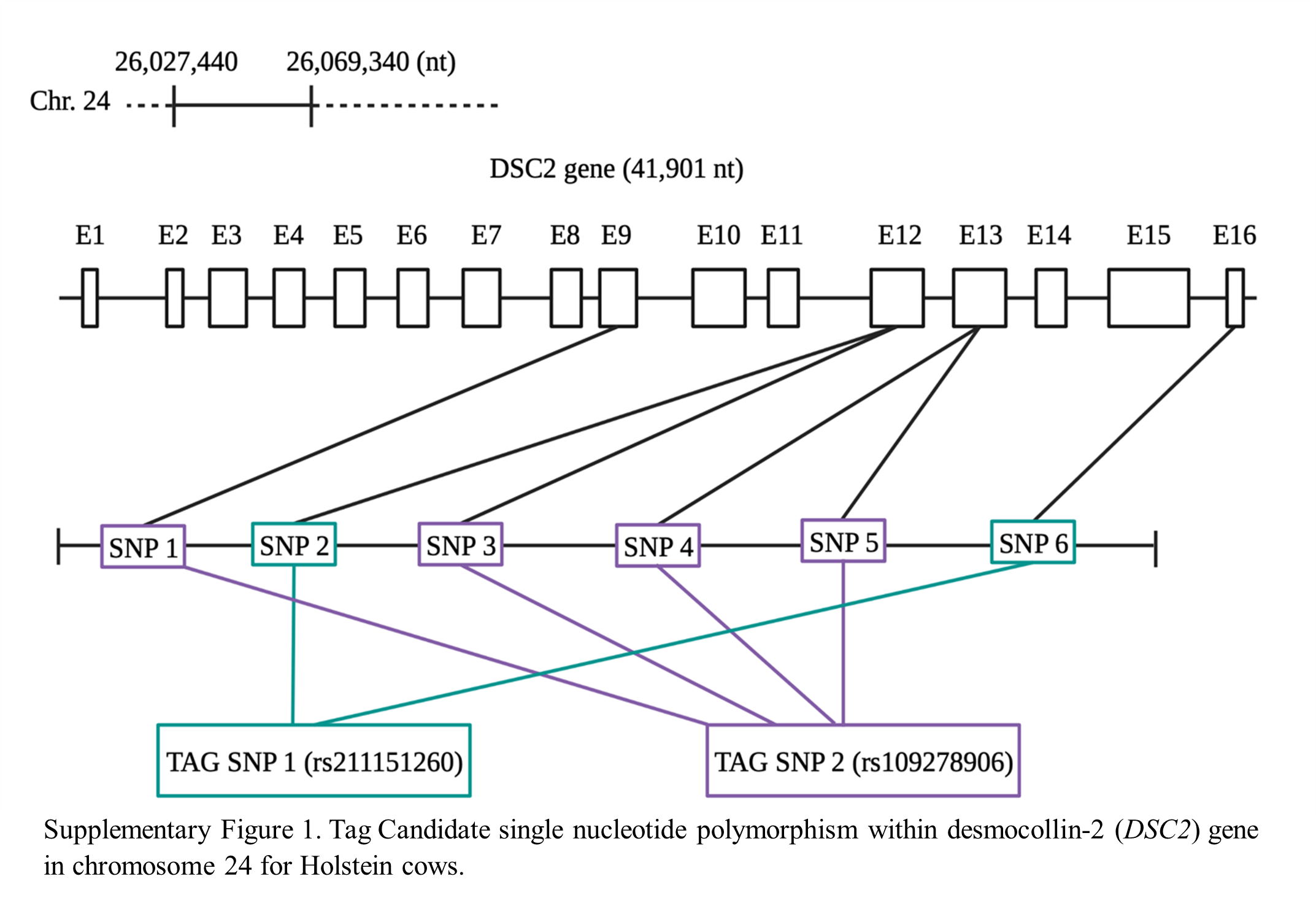

Supplement: Supplementary file 2 [file Image1.tif]
