# Supplementary material for: Identification of candidate SNPs associated with embryo mortality and fertility traits in lactating Holstein cows
Source: Front Genet. 2024 Aug 9;15:1409335. doi: 10.3389/fgene.2024.1409335 (PMC11341358; doi:10.3389/fgene.2024.1409335)
Supplement: Supplementary file 3 [file Table2.DOCX]

| **Supplementary table 2.** Summary of conception, age at calving and pregnancy with candidate single nucleotide polymorphism (SNP; n=17) in primiparous lactating Holstein cows. | | | | | | | |
| --- | --- | --- | --- | --- | --- | --- | --- |
| **Trait** | **Gene** | **RSID^1^** | **n** | **Model**  **P-value** | **Model**  **X^2^ Test**  **P-value** | **SNP**  **P-value** | **SNP**  **X^2^ Test**  **P-value** |
| Services per conception^a^ |  |  |  |  |  |  |  |
|  | *UMPS* | rs110953962 | 463 | 0.002 | . | 0.2 | . |
|  | *HSD17B7* | rs110828053 | 464 | 0.001 | . | 0.1 | . |
|  | *CAST* | rs110914810 | 466 | 0.006 | . | 0.9 | . |
|  | *ACAT2* | rs109967779 | 464 | 0.005 | . | 0.6 | . |
|  | *IFNGR1* | rs109049057 | 466 | 0.004 | . | 0.6 | . |
|  | *DECR1* | rs41580472 | 465 | 0.003 | . | 0.3 | . |
|  | *MRPL48* | . | 466 | 0.002 | . | 0.2 | . |
|  | *FASN* | rs41919985 | 433 | 0.005 | . | 0.3 | . |
|  | *SREBF1* | rs41912290 | 465 | 0.001 | . | 0.1 | . |
|  | *BOLA-DMB* | rs109032590 | 464 | 0.004 | . | 0.5 | . |
|  | *BLA-DQB* | rs109291107 | 409 | 0.004 | . | 0.5 | . |
|  | *BOLA-NC1* | rs382125666 | 401 | 0.006 | . | 0.2 | . |
|  | *UBD* | rs209518868 | 465 | 0.006 | . | 0.8 | . |
|  | *UBD* | rs109295136 | 400 | 0.002 | . | 0.09† | . |
|  | *DSC2* | rs109300814 | 463 | 0.005 | . | 0.4 | . |
|  | *DSC2* | rs211151260 | 466 | 0.007 | . | 0.9 | . |
|  | *DSC2* | rs109278906 | 463 | 0.005 | . | 0.6 | . |
| Age at 1^st^ calving^a^ |  |  |  |  |  |  |  |
|  | *UMPS* | rs110953962 | 463 | 0.2 | . | 0.4 | . |
|  | *HSD17B7* | rs110828053 | 464 | 0.4 | . | 0.8 | . |
|  | *CAST* | rs110914810 | 466 | 0.4 | . | 0.9 | . |
|  | *ACAT2* | rs109967779 | 464 | 0.2 | . | 0.5 | . |
|  | *IFNGR1* | rs109049057 | 466 | 0.2 | . | 0.4 | . |
|  | *DECR1* | rs41580472 | 465 | 0.2 | . | 0.3 | . |
|  | *MRPL48* | . | 466 | 0.2 | . | 0.4 | . |
|  | *FASN* | rs41919985 | 433 | 0.1 | . | 0.07 | . |
|  | *SREBF1* | rs41912290 | 465 | 0.2 | . | 0.4 | . |
|  | *BOLA-DMB* | rs109032590 | 464 | 0.1 | . | 0.1 | . |
|  | *BLA-DQB* | rs109291107 | 409 | 0.3 | . | 0.9 | . |
|  | *BOLA-NC1* | rs382125666 | 401 | 0.2 | . | 0.4 | . |
|  | *UBD* | rs209518868 | 465 | 0.3 | . | 0.8 | . |
|  | *UBD* | rs109295136 | 400 | 0.3 | . | 0.8 | . |
|  | *DSC2* | rs109300814 | 463 | 0.05 | . | 0.04* | . |
|  | *DSC2* | rs211151260 | 466 | 0.04 | . | 0.03* | . |
|  | *DSC2* | rs109278906 | 463 | 0.3 | . | 0.7 | . |
| Days to conception^a^ |  |  |  |  |  |  |  |
|  | *UMPS* | rs110953962 | 416 | 0.01 | . | 0.3 | . |
|  | *HSD17B7* | rs110828053 | 415 | 0.01 | . | 0.2 | . |
|  | *CAST* | rs110914810 | 417 | 0.03 | . | 0.9 | . |
|  | *ACAT2* | rs109967779 | 415 | 0.02 | . | 0.5 | . |
|  | *IFNGR1* | rs109049057 | 417 | 0.01 | . | 0.3 | . |
|  | *DECR1* | rs41580472 | 416 | 0.02 | . | 0.6 | . |
|  | *MRPL48* | . | 417 | 0.04 | . | 0.9 | . |
|  | *FASN* | rs41919985 | 388 | 0.04 | . | 0.5 | . |
|  | *SREBF1* | rs41912290 | 416 | 0.003 | . | 0.04* | . |
|  | *BOLA-DMB* | rs109032590 | 415 | 0.007 | . | 0.1 | . |
|  | *BLA-DQB* | rs109291107 | 367 | 0.03 | . | 0.3 | . |
|  | *BOLA-NC1* | rs382125666 | 358 | 0.01 | . | 0.5 | . |
|  | *UBD* | rs209518868 | 416 | 0.001 | . | 0.009* | . |
|  | *UBD* | rs109295136 | 361 | 0.05 | . | 0.1 | . |
|  | *DSC2* | rs109300814 | 414 | 0.02 | . | 0.2 | . |
|  | *DSC2* | rs211151260 | 417 | 0.03 | . | 0.9 | . |
|  | *DSC2* | rs109278906 | 414 | 0.01 | . | 0.1 | . |
| Days to 1^st^ AI^a^ |  |  |  |  |  |  |  |
|  | *UMPS* | rs110953962 | 463 | <.0001 | . | 0.04* | . |
|  | *HSD17B7* | rs110828053 | 464 | <.0001 | . | 0.06† | . |
|  | *CAST* | rs110914810 | 466 | <.0001 | . | 0.6 | . |
|  | *ACAT2* | rs109967779 | 464 | <.0001 | . | 0.1 | . |
|  | *IFNGR1* | rs109049057 | 466 | <.0001 | . | 0.7 | . |
|  | *DECR1* | rs41580472 | 465 | <.0001 | . | 0.3 | . |
|  | *MRPL48* | . | 466 | <.0001 | . | 0.2 | . |
|  | *FASN* | rs41919985 | 433 | <.0001 | . | 0.07† | . |
|  | *SREBF1* | rs41912290 | 465 | <.0001 | . | 0.01* | . |
|  | *BOLA-DMB* | rs109032590 | 464 | <.0001 | . | 0.08† | . |
|  | *BLA-DQB* | rs109291107 | 409 | <.0001 | . | 0.06† | . |
|  | *BOLA-NC1* | rs382125666 | 401 | <.0001 | . | 0.7 | . |
|  | *UBD* | rs209518868 | 465 | <.0001 | . | 0.4 | . |
|  | *UBD* | rs109295136 | 400 | 0.002 | . | 0.7 | . |
|  | *DSC2* | rs109300814 | 463 | <.0001 | . | 0.1 | . |
|  | *DSC2* | rs211151260 | 466 | <.0001 | . | 0.5 | . |
|  | *DSC2* | rs109278906 | 463 | <.0001 | . | 0.5 | . |
| Pregnant at 1^st^ AI^b^ |  |  |  |  |  |  |  |
|  | *UMPS* | rs110953962 | 463 | . | 0.1 | . | 0.3 |
|  | *HSD17B7* | rs110828053 | 464 | . | 0.1 | . | 0.6 |
|  | *CAST* | rs110914810 | 466 | . | 0.2 | . | 0.8 |
|  | *ACAT2* | rs109967779 | 464 | . | 0.1 | . | 0.6 |
|  | *IFNGR1* | rs109049057 | 466 | . | 0.2 | . | 0.7 |
|  | *DECR1* | rs41580472 | 465 | . | 0.02 | . | 0.02* |
|  | *MRPL48* | . | 466 | . | 0.1 | . | 0.4 |
|  | *FASN* | rs41919985 | 433 | . | 0.01 | . | 0.02* |
|  | *SREBF1* | rs41912290 | 465 | . | 0.1 | . | 0.4 |
|  | *BOLA-DMB* | rs109032590 | 464 | . | 0.09 | . | 0.1 |
|  | *BLA-DQB* | rs109291107 | 409 | . | 0.08 | . | 0.9 |
|  | *BOLA-NC1* | rs382125666 | 401 | . | 0.2 | . | 0.5 |
|  | *UBD* | rs209518868 | 465 | . | 0.1 | . | 0.8 |
|  | *UBD* | rs109295136 | 400 | . | 0.04 | . | 0.03 |
|  | *DSC2* | rs109300814 | 463 | . | 0.1 | . | 0.3 |
|  | *DSC2* | rs211151260 | 466 | . | 0.1 | . | 0.2 |
|  | *DSC2* | rs109278906 | 463 | . | 0.1 | . | 0.3 |
| Pregnant at 150 DIM^b^ |  |  |  |  |  |  |  |
|  | *UMPS* | rs110953962 | 463 | . | 0.006 | . | 0.1 |
|  | *HSD17B7* | rs110828053 | 464 | . | 0.01 | . | 0.3 |
|  | *CAST* | rs110914810 | 466 | . | 0.02 | . | 0.8 |
|  | *ACAT2* | rs109967779 | 464 | . | 0.01 | . | 0.3 |
|  | *IFNGR1* | rs109049057 | 466 | . | 0.01 | . | 0.2 |
|  | *DECR1* | rs41580472 | 465 | . | 0.003 | . | 0.06† |
|  | *MRPL48* | . | 466 | . | 0.01 | . | 0.4 |
|  | *FASN* | rs41919985 | 433 | . | 0.02 | . | 0.3 |
|  | *SREBF1* | rs41912290 | 465 | . | 0.001 | . | 0.01* |
|  | *BOLA-DMB* | rs109032590 | 464 | . | 0.002 | . | 0.04* |
|  | *BLA-DQB* | rs109291107 | 409 | . | 0.02 | . | 0.9 |
|  | *BOLA-NC1* | rs382125666 | 401 | . | 0.01 | . | 0.8 |
|  | *UBD* | rs209518868 | 465 | . | 0.01 | . | 0.6 |
|  | *UBD* | rs109295136 | 400 | . | 0.07 | . | 0.1 |
|  | *DSC2* | rs109300814 | 463 | . | 0.009 | . | 0.1 |
|  | *DSC2* | rs211151260 | 466 | . | 0.03 | . | 0.9 |
|  | *DSC2* | rs109278906 | 463 | . | 0.008 | . | 0.1 |
| Pregnancy loss^b^ |  |  |  |  |  |  |  |
|  | *UMPS* | rs110953962 | 463 | . | 0.7 | . | 0.7 |
|  | *HSD17B7* | rs110828053 | 464 | . | 0.3 | . | 0.1 |
|  | *CAST* | rs110914810 | 466 | . | 0.7 | . | 0.6 |
|  | *ACAT2* | rs109967779 | 464 | . | 0.07 | . | 0.05† |
|  | *IFNGR1* | rs109049057 | 466 | . | 0.1 | . | 0.01 |
|  | *DECR1* | rs41580472 | 466 | . | 0.5 | . | 0.4 |
|  | *MRPL48* | . | 466 | . | 0.7 | . | 0.8 |
|  | *FASN* | rs41919985 | 433 | . | 0.4 | . | 0.3 |
|  | *SREBF1* | rs41912290 | 465 | . | 0.4 | . | 0.1 |
|  | *BOLA-DMB* | rs109032590 | 464 | . | 0.6 | . | 0.4 |
|  | *BLA-DQB* | rs109291107 | 409 | . | 0.5 | . | 0.3 |
|  | *BOLA-NC1* | rs382125666 | 401 | . | 0.7 | . | 0.4 |
|  | *UBD* | rs209518868 | 465 | . | 0.7 | . | 0.7 |
|  | *UBD* | rs109295136 | 400 | . | 0.7 | . | 0.8 |
|  | *DSC2* | rs109300814 | 463 | . | 0.7 | . | 0.7 |
|  | *DSC2* | rs211151260 | 466 | . | 0.7 | . | 0.7 |
|  | *DSC2* | rs109278906 | 463 | . | 0.7 | . | 0.7 |
| ^a^Non-binary models that were evaluated with GLM-one way ANOVA. ^b^Binary models that were evaluated with logistic regression. †Candidate single nucleotide polymorphism that were a tendency to be significant (P<0.10) within models. *Candidate single nucleotide polymorphism that were statistically significant (P<0.05) within models. RSID^1^=reference SNP identification. AI=artificial insemination; DIM= days in milk. | | | | | | | |
