# Supplementary material for: Identification of candidate SNPs associated with embryo mortality and fertility traits in lactating Holstein cows
Source: Front Genet. 2024 Aug 9;15:1409335. doi: 10.3389/fgene.2024.1409335 (PMC11341358; doi:10.3389/fgene.2024.1409335)
Supplement: Supplementary file 4 [file Table3.docx]

| **Supplementary table 3.** Genes nearby or associated with reproductive/fertility traits within the Cattle Quantitative Trait Loci Data Base (CattleQTLdb). | | |  |
| --- | --- | --- | --- |
| **Reproductive/**  **Fertility Trait** | **Gene** | **Cattle QTLdb Link** |  |
| Days to Conception | CAST | <https://www.animalgenome.org/cgi-bin/QTLdb/BT/qdetails?QTL_ID=127047> |  |
|  | BOLA-DMB | <https://www.animalgenome.org/cgi-bin/QTLdb/BT/qdetails?QTL_ID=126888> |  |
|  | BOLA-DQB | <https://www.animalgenome.org/cgi-bin/QTLdb/BT/qdetails?QTL_ID=126888> |  |
|  | BOLA-NCI | <https://www.animalgenome.org/cgi-bin/QTLdb/BT/qdetails?QTL_ID=126888> |  |
|  | UBD | <https://www.animalgenome.org/cgi-bin/QTLdb/BT/qdetails?QTL_ID=126888> |  |
| Pregnant at first AI | UMPS | <https://www.animalgenome.org/cgi-bin/QTLdb/BT/qdetails?QTL_ID=176668>  <https://www.animalgenome.org/cgi-bin/QTLdb/BT/qdetails?QTL_ID=176669> |  |
|  | HSD17B7 | <https://www.animalgenome.org/cgi-bin/QTLdb/BT/qdetails?QTL_ID=127019> |  |
|  | DECR1 | <https://www.animalgenome.org/cgi-bin/QTLdb/BT/qdetails?QTL_ID=176803> |  |
|  | MRLP48 | <https://www.animalgenome.org/cgi-bin/QTLdb/BT/qdetails?QTL_ID=176837> |  |
|  | SREBF1 | <https://www.animalgenome.org/cgi-bin/QTLdb/BT/qdetails?QTL_ID=176837> |  |
|  | FASN | <https://www.animalgenome.org/cgi-bin/QTLdb/BT/qdetails?QTL_ID=22894> |  |
|  | BOLA-DQB1  and UBD | <https://www.animalgenome.org/cgi-bin/QTLdb/BT/qdetails?QTL_ID=176854> <https://www.animalgenome.org/cgi-bin/QTLdb/BT/qdetails?QTL_ID=212359> |  |
|  | DSC2 | <https://www.animalgenome.org/cgi-bin/QTLdb/BT/qdetails?QTL_ID=212367> |  |
| Conception Rate | IFNGR1 | <https://www.animalgenome.org/cgi-bin/QTLdb/BT/qdetails?QTL_ID=181260> |  |
|  | ACAT2 | <https://www.animalgenome.org/cgi-bin/QTLdb/BT/qdetails?QTL_ID=177083> | |
|  | MRPL48 | <https://www.animalgenome.org/cgi-bin/QTLdb/BT/qdetails?QTL_ID=181330> | |
